# *PPP2R5D*-Related Neurodevelopmental Disorder and Multiple Haemangiomas: A Novel Phenotypic Trait?

**DOI:** 10.3390/pediatric16040101

**Published:** 2024-12-16

**Authors:** Francesco Comisi, Consolata Soddu, Francesco Lai, Monica Marica, Michela Lorrai, Giancarlo Mancuso, Sabrina Giglio, Salvatore Savasta

**Affiliations:** 1 Pediatrics Department, Microcitemico Hospital “A. Cao”, University of Cagliari, 09124 Cagliari, Italy; francescocomisi@virgilio.it; 2 Pediatric and Rare Diseases Clinic, Microcitemico Hospital “A. Cao”, ASL 8 Cagliari, 09121 Cagliari, Italy; consolata.soddu@aob.it (C.S.); monica.marica@aob.it (M.M.); 3 Unit of Oncology and Molecular Pathology, Department of Biomedical Sciences, University of Cagliari, 09124 Cagliari, Italy; 4 Medical Genetics Unit, Department of Medical Sciences and Public Health, University of Cagliari, 09124 Cagliari, Italy; michelalorrai9@gmail.com (M.L.); giancarlomancuso5@gmail.com (G.M.); sabrinar.giglio@unica.it (S.G.); 5 Centre for Research University Services, University of Cagliari, 09124 Cagliari, Italy; 6 Medical Genetics, “R. Binaghi” Hospital, ASL 8 Cagliari, 09126 Cagliari, Italy; 7 Pediatric and Rare Diseases Clinic, Microcitemico Hospital “A. Cao”, Department of Medical Sciences and Public Health, University of Cagliari, 09124 Cagliari, Italy; salvatore.savasta@unica.it

**Keywords:** haemangiomas, PPP2R5D, Jordan’s syndrome, PI3K/AKT signalling pathway

## Abstract

Background: Houge-Janssens syndrome 1 is a condition with onset in early childhood caused by heterozygous pathogenic variants in the *PPP2R5D* gene, which encodes a B56 regulatory subunit of the serine/threonine protein phosphatase 2A (PP2A). There is evidence that the PP2A-PPP2R5D complex is involved in regulating the phosphatidylinositol 3-kinase (PI3K)/AKT signalling pathway, which is crucial for several cellular processes, including the pathogenesis and progression of haemangiomas. Case presentation: We report the first *PPP2R5D*-related neurodevelopmental disorder case from Sardinia, a child with transient hypoglycaemia, facial dysmorphisms, and multiple haemangiomas. Whole Exome Sequencing analysis confirmed the clinical suspicion, detecting the presence of the de novo missense variant c.592G>A in the *PPP2R5D* gene. Conclusions: Haemangiomas have never been linked to the syndromic phenotype of the *PPP2R5D*-associated disorder. The close correlation between the PP2A enzyme and the PI3K/AKT signalling pathway suggests the possible correlation between its dysfunction and activation of haemangiogenesis. Our report highlights a possible link between the PPP2R5D-related disorder and altered angiogenesis, characterizing diffuse haemangiomas as a possible novel phenotypic trait of this condition.

## 1. Introduction

The *PPP2R5D* (Protein Phosphatase 2 Regulatory Subunit B’ Delta)-related disorder, also known as Houge–Janssens syndrome 1 (OMIM #616355) or Jordan’s syndrome, is an autosomal, dominant (AD) condition with onset in early childhood characterized by moderate-to-severe developmental delays. It was first described by Houge et al. in 2015; to date, 100 cases have been reported [1,2]. Distinctive clinical features include intellectual disability, speech impairment, seizures, macrocephaly, autism spectrum disorders, generalized hypotonia with delayed motor skill development, facial dysmorphisms (hypertelorism, the down slanting of palpebral fissures, a broad, tall forehead, hypotonic facies, low-set ears), and skeletal, endocrine, cardiac, and genital abnormalities [1,3,4]. Hydrocephalus, ventricular dilation, partial or complete corpus callosum agenesis, cavum septum pellucidum, and white matter anomalies are nonspecific brain MRI findings [1]. The *PPP2R5D*-related disorder is caused by heterozygous de novo pathogenetic variants in the *PPP2R5D* gene (OMIM * 601646) on chromosome 6p21 [1,5,6]. This gene encodes the B56δ subunit of the PPP2R5D protein, an isoform of the serine/threonine protein phosphatase 2A (PP2A), highly expressed in the human brain [7,8]. The heteromeric enzyme PP2A is characterized by three distinct subunits: a 65 kDa scaffolding subunit A, a regulatory subunit B, and a 36 kDa catalytic subunit C [9]. This enzyme performs various substrate-specific physiological functions in different tissues [10,11]. The severity of intellectual disability and neurodevelopmental delay seems to be linked to the magnitude of the biochemical impairment in PP2A activity [4]. PP2A also plays a crucial role in several signalling pathways, particularly those regulated by reversible phosphorylation [12,13]. There is evidence that PP2A-PPP2R5D is involved in regulating the phosphatidylinositol 3-kinase (PI3K)/AKT signalling pathway, which is fundamental in neuronal maintenance [14] and in multiple cellular functions [15]. Furthermore, the PI3K/AKT signalling cascade is essential for normal blood vessel development during embryogenesis [16]. The main aim of this paper is to identify a possible link between the *PPP2R5D*-related disorder and altered angiogenesis, characterising diffuse haemangiomas as a novel phenotypic trait of this syndrome in a child with transient hypoglycaemia, facial dysmorphisms, and multiple haemangiomas.

## 2. Case Presentation

**Figure 1:**
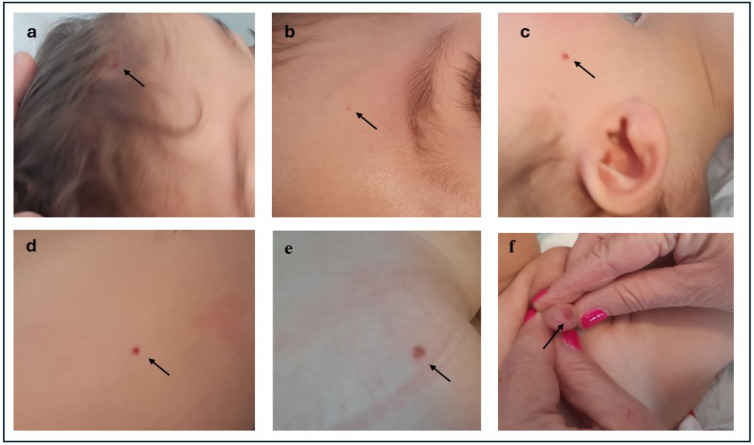
Multiple haemangiomas: (**a**) flat forehead haemangioma; (**b**) small, flat eyebrow haemangioma; (**c**) small, raised right cheek haemangioma; (**d**) small, raised abdominal haemangioma; (**e**) flat right glute haemangioma; and (**f**) large, raised penile haemangioma.

The patient is a 1-year-old child born to non-consanguineous parents after a normal pregnancy and delivery at 39 weeks of gestation. The couple has a history of recurrent pregnancy loss, with five first-trimester miscarriages preceding this pregnancy, but no family history of haemangiomas or similar findings across three generations was identified. The pregnancy was carefully monitored, including first-trimester biochemical testing and a non-invasive prenatal test (NIPT), which showed no evidence of chromosomal aneuploidies. Serial gynecological ultrasounds throughout gestation revealed no abnormalities. At birth, the infant had normal growth parameters, with a birth weight of 3280 g, length of 51 cm, and a head circumference of 37 cm, combined with a normal Apgar score. The perinatal period was characterised by transient hypoglycaemia and metabolic acidosis, requiring admission to the Neonatal Pathology department. At admission, the infant’s blood glucose level was recorded at 35 mg/dL. Over the following hours, the levels progressively increased and stabilised, with subsequent readings of 46 mg/dL at two hours, 58 mg/dL at four hours, 62 mg/dL at six hours, and 70 mg/dL at nine hours. An initial clinical evaluation revealed a constellation of dysmorphic features, including macrocephaly with a wide anterior fontanel, frontal bossing, low-set ears, telecanthus, the down-slanting of palpebral fissures, a depressed nasal bridge, a deep philtrum, a cupid bow upper lip, an asymmetric jaw with left hypoplasia, micrognathia, pectus excavatum, skin laxity, and joint hypermobility. Doppler echocardiography identified a patent ductus arteriosus (PDA) with left-to-right shunting and an aneurysmatic interatrial septum, both of which were resolved by discharge. Extensive evaluations, including blood tests, brain MRI, EEG, auditory brainstem response (ABR), and transabdominal ultrasound, did not evidence any pathological signs. Postnatally, a strict follow-up regimen was started. At three months of age, the patient underwent surgery for an inguinal hernia and the excision of a chest angioma. The histological examination of the excised lesion revealed a pathological diagnosis of lobular capillary haemangioma. Regarding psychomotor developmental milestones, the child exhibited vocalizations at three months but still did not produce words or sentences at 12 months. Eye contact and social interactions were remarkably good from infancy. The acquisition of motor milestones is globally delayed: the patient started to roll sideways at eight months but has not yet developed sitting, walking with support from furniture objects or independent walking. At 12 months, persistent axial hypotonia, epilepsy, and stereotypes were observed, as well as several diffuse, bright, red haemangiomas angiomas of different sizes and volumes, both flat and raised. Haemangiomas are of millimetric dimensions; the two biggest haemangiomas are located on the abdomen (5 × 4 mm) and the other on the glans penis (14 × 9 mm) (Figure 1).

Whole Exome Sequencing (WES) was carried out to establish a molecular diagnosis. Genomic DNA was isolated from the patient’s peripheral blood sample using the QIAamp^®^ DNA Blood Kit (Qiagen, Hilden, Germany) in accordance with the manufacturer’s instructions. Library preparation was conducted using the KAPA HyperExome Plus Kit, KAPA Universal Adapter, and KAPA HyperCapture Bead Kit, following the KAPA HyperCap Workflow v3.0 protocol (Roche Sequencing and Life Science, Boston, MA, USA). Sequencing analysis was conducted on an Illumina NextSeq550 platform (Illumina Inc., San Diego, CA, USA). Variants identified in candidate genes were filtered by comparing them against widely recognised population databases (gnomAD, ExAC, and the 1000 Genomes Project), excluding those with allelic frequencies greater than 0.1% in the European Non-Finnish population. The clinical relevance of the variants was assessed following the 2015 guidelines established by the American College of Medical Genetics and Genomics (ACMG) [17]. WES identified the de novo heterozygous missense variant c.592G>A (p. Glu198Lys) in the *PPP2R5D* gene (NM_006245.4), responsible for Houge–Janssens syndrome 1. This variant leads to the substitution of Glutamic Acid with Lysine at position 198 in the peptide chain. It is reported as rs863225082 in the dbSNP database but is absent in the gnomAD sequencing database. Notably, it is listed in the ClinVar database (Access. No.: VCV000190286.64) as a pathogenic variant. The c.592G>A substitution affects an amino acid that is evolutionarily conserved across species and is predicted to be probably damaging by the majority of bioinformatics tools consulted. Following ACMG guidelines, this variant is classified as pathogenic. Heterozygous pathogenic/likely pathogenic variants in *GJB2*, *RPE65*, and *CD36* genes were identified, which did not correlate with the patient’s phenotype or the presence of haemangiomas.

## 3. Discussion

PP2A is recognised for its essential role in numerous cellular functions, where it governs cell metabolism and oversees various mechanisms, including the cell cycle, DNA replication, transcription, translation, cell proliferation, and intracellular signalling [18]. PP2A exists as a trimeric holoenzyme complex. The trimeric structure is composed of three subunits: catalytic, scaffold, and regulatory subunits [18,19]. Regulatory subunits are divided into four distinct families: B (B55/PR55), B′ (B56/PR 61), B″ (PR48/PR72/PR 130), and B‴ (PR93/PR110). Previous research suggests that the B56 subunit, encoded by the *PPP2R5D* gene, plays a key role in several functions, including the negative regulation of the PI3K/AKT growth regulatory pathway [18]. The regulation of vasculature and angiogenesis involves multiple pathways, among which the one mediated by PI3K/AKT plays a crucial role [20]. Jiang et al. were the first to provide direct evidence of PI3K and AKT’s contribution to the regulation of angiogenesis in vivo, utilising the RCAS retroviral vector system to induce the expression of these proteins [21]. Among the three isoforms of AKT, AKT1 is predominant in vascular cells and closely related to vasculature during animal development and pathological angiogenesis, as demonstrated by Yang and colleagues [20,21,22]. In 2011, Karar and Maity showed that the modulation of nitric oxide and angiopoietins expression carried out by the PI3K/AKT/mTOR cascade can affect angiogenesis [16]. Furthermore, Ji et al. reported that this signalling pathway is involved in the development and progression of haemangioma [23].

The overactivation of PI3K/AKT signalling is a common feature of the majority of low-flow vascular malformations [24,25,26]. Gain-of-function mutations, resulting in the hyperactivation of the endothelial receptor tyrosine kinase or the PI3K alpha catalytic subunit PIK3CA, cause the formation of vascular malformations, particularly of the sporadic venous malformation type [27,28]. Additionally, venous and/or capillary malformations frequently occur in overgrowth syndromes associated with PIK3CA pathogenic variants, known collectively as the PIK3CA-related overgrowth spectrum (PROS) [29,30,31].

Haemangioma is a benign vascular neoplasm derived from strongly over-proliferative endothelial cell growth. The pathogenesis of haemangiomas has not yet been completely defined. In 2015, Xie and colleagues were the first to demonstrate the anti-proliferative effect of PP2A in vascular endothelial cells. They generated an endothelial-specific PyMT (polyoma middle T antigen) gene-expressing transgenic mouse that spontaneously developed haemangiomas. A distinct interaction between PyMT and PP2A was detected in endothelial cells expressing PyMT, resulting in the separation of the B subunit from the PP2A complex and the subsequent deactivation of PP2A phosphatases. The inactivation of PP2A phosphatase upstream, along with the phosphorylation of AKT and ERK downstream, resulted in an increased replication rate and enhanced migratory and angiogenic capacities of vascular endothelial cells in vitro. In vivo, this led to an increased capacity for tumorigenesis [32].

To our knowledge, haemangiomas have never been linked to the syndromic phenotype of the *PPP2R5D*-associated disorder. As already indicated, this is an AD-transmitted intellectual developmental disease of which, from the review of the literature, only one other case has been described showing these common vascular benign tumours: a 7-month-old patient presented with two haemangiomas on the lower lip and back (Table 1) [33].

The genomic complexity of *PPP2R5D*-related disorders is underscored by the gene’s role in diverse cellular functions and its evolutionary conservation. Variants often occur in hotspots which affect highly conserved amino acids critical for PP2A binding and function, such as the variant detected in our patient. These alterations lead to pleiotropic effects on neuronal development, manifesting as a spectrum of clinical features, including intellectual disability, developmental delay, hypotonia, and dysmorphic features. The complexity is further amplified by variable expressivity and the interplay of modifier genes and environmental factors, which contribute to the heterogeneous phenotype observed among affected individuals [4].

No association was made between the syndrome and the presence of haemangiomas.

It is reasonable to hypothesise that the structural alteration of the PP2A enzyme in the *PPP2R5D*-related disorder may lead to an activation of the PI3K/AKT signalling pathway, promoting haemangiomagenesis.

## 4. Conclusions

Our report may provide new perspectives with which to understand *PPP2R5D* genotype–phenotype correlations. Haemangiomas may represent a novel *PPP2R5D*-related disorder phenotypic trait, expanding the clinical spectrum of this condition. Further studies are needed in order to conclude this potential causal association, better understand the pathophysiology of this condition, and identify potential therapeutic targets.

## Figures and Tables

**Table 1:** Summary of the clinical features of reported cases with the molecular diagnosis of the *PPP2R5D*-related disorder and multiple haemangiomas. HC: head circumference; SD: standard deviations; MRI: Magnetic Resonance Imaging.

	Our Case	Maines et al. [33]
Head Circumference	Macrocephaly with HC 37 cm (2.3 SD)	Macrocephaly with HC 47.5 cm (4.1 SD)
Dysmorphic Features	Frontal bossing and telecanthus	Wide forehead
	Down-slanting palpebral fissures	Epicanthus
	Depressed nasal bridge	
	Deep philtrum Cupid bow on the upper lip	
	Asymmetric jaw with left hypoplasia	
	Micrognathia	
	Pectus excavatum	
Haemangiomas	Several diffuse, bright red haemangiomas of various sizes and types at 12 months.	Two haemangiomas (lip, back) at 7 months
Neurological Findings	Persistent axial hypotonia	Nystagmus
	Epilepsy	Seizures
	Stereotypes	Developmental delay
		Diffuse hypotonia
Hypoglycaemia	Transient hypoglycaemia at birth	Hypoglycaemic episodes in the first days of life
MRI Findings	Normal brain MRI	Normal brain MRI

## Data Availability

The original contributions presented in the study are included in the article; further inquiries can be directed to the corresponding author.
